# Integrated Strategies Toward the Capture and Electrochemical Conversion of Low‐Concentration Carbon Dioxide

**DOI:** 10.1002/EXP.20240006

**Published:** 2025-05-04

**Authors:** Zhenyi Yang, Xingqiu Li, Xianglong Cui, Zhen Zheng, Penglun Zheng, Yu Zhang

**Affiliations:** ^1^ School of Mechanical and Power Engineering East China University of Science and Technology Shanghai China; ^2^ Department of Materials Science & Engineering National University of Singapore Singapore Singapore; ^3^ Civil Aircraft Fire Science and Safety Engineering Key Laboratory of Sichuan Province College of Civil Aviation Safety Engineering Civil Aviation Flight University of China, Sichuan Guanghan China

**Keywords:** carbon capture, CO_2_ electrolysis, electrocatalyst, integrated strategy, system

## Abstract

Electrochemical reduction of carbon dioxide (CO_2_) has been considered a promising route to reduce net carbon emissions and thus mitigate global warming issues. In practice, it mainly involves two processes including the CO_2_ capture and subsequent electrochemical conversion. From the perfective of feasible and economic benefits, it is of practical significance to develop integrated CO_2_ capture and conversion systems in an efficient way. However, a majority of studies have been currently focusing on the independent process, and the development of integrated strategies is still in the initial stage. This review mainly covers the recent progress on the integrated technologies of CO_2_ capture and electrochemical conversion, including the integration strategies, mechanisms, and corresponding issues. The advantages and disadvantages of those strategies are particularly discussed, aiming to identify the viable routes for future applications. To conclude, the challenges and prospects in terms of the research direction in this field are provided, with the hope of promoting practical CO_2_ utilization from the fundamental aspects.

## Graphical abstract

This review mainly covers the recent progress on the integrated technologies of CO_2_ capture and electrochemical conversion, including the integration strategies, mechanisms, and corresponding issues. The challenges and prospects in terms of the research direction in this field are also provided, with the hope of promoting practical CO_2_ utilization from the fundamental aspects.

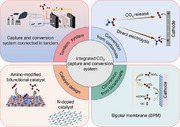

## 1 Introduction

The massive CO_2_ emission is motivating the development of various carbon capture, utilization, and storage (CCUS) technologies in response to global warming and energy crisis issues [1, 2, 3, 4, 5]. However, challenges remain for widely studied CO_2_ storage underground or in the deep ocean due to the potential leakage. In this regard, direct CO_2_ utilization is considered an effective route in the long term relative to temporary storage [6]. Particularly, converting CO_2_ into valuable fuels is beneficial for simultaneously reducing net emissions and providing sustainable energy [7, 8, 9, 10]. At present, CO_2_ conversion technologies mainly include biochemical, photochemical, electrochemical, and thermo‐electrochemical catalytic methods [11, 12, 13, 14, 15, 16]. In particular, the electrochemical CO_2_ reduction reaction (CO_2_RR) has garnered significant attention due to its merits of high efficiency, favorable working conditions, and ease of scale [17, 18, 19]. Several types of reductive products (e.g., CO, HCOOH or HCOO^−^, CH_4_, C_2_H_4_, C_2_H_5_OH, CH_3_COOH) can be selectively produced by utilizing the specific electrocatalysts, demonstrating the wide application potentials of CO_2_RR [20, 21, 22, 23].

Considering the limited solubility of CO_2_ in aqueous solutions, CO_2_RR has been mainly carried out using high‐purity CO_2_ gas (>99.99%) at a laboratory scale to evaluate the performance of catalysts or devices [24]. However, the production of high‐purity CO_2_ requires the capture processes from dilute sources (e.g., air, ocean) or point sources (e.g., flue gases from power plants or refineries). The process of releasing, compressing, and transporting CO_2_ from the capture agent requires a large amount of energy input, greatly increasing the cost of carbon capture [25, 26, 27]. To this end, integrating the low‐concentration CO_2_ capture and conversion, which are carried out simultaneously, can be beneficial for simplifying the procedures and thus reduce the investment cost [28, 29, 30, 31, 32]. Previous studies have demonstrated that directly coupling CO_2_ capture and electrochemical conversion technology may save nearly 44% of energy consumption and 21% of energy costs compared to the most advanced gas‐fed CO_2_ electrolyzer [33]. During the whole process, the capture fluid of CO_2_ serves as a carrier for transporting CO_2_ to the electrochemical reactors, which is then regenerated after the CO_2_ conversion [34]. However, the integrated technologies suffer from lower CO_2_ concentration relative to the employment of high‐purity CO_2_, leading to inferior mass transportation and competing hydrogen evolution reaction (HER). Therefore, it is still highly desirable to develop cost‐effective strategies for integrating CO_2_ capture and conversion, aiming to be directly employed in heavy‐emission plants.

In this review, the integrated strategies are mainly divided into two categories, including the tandem connection of capture and conversion systems and the direct conversion of CO_2_‐capturing solution. For the tandem connection, recent advances in CO_2_ capture technologies are emphasized, since the following conversion step is similar to the usage of high‐purity CO_2_ gas. For the direct conversion, the latest progress based on different capture solutions is overviewed and discussed, covering the corresponding mechanisms and remaining issues. In addition, a special focus is also given to the design of catalysts that can simultaneously adsorb and convert CO_2_, which is another emerging feasible and promising method. Finally, the challenges and future directions of integrated CO_2_ capture and conversion technologies are presented.

## 2 Tandem Connection

Connecting CO_2_ capture and conversion systems in tandem is a straightforward strategy, both of which occur independently. In this configuration, the CO_2_ gas is initially captured from a dilute or point source and then released with higher concentrations as an input to the subsequent conversion system. Simultaneously, the capture agent can be regenerated. According to the working mechanism, the tandem connection between two separate processes provides significant flexibility. A capture system with high efficiency and low energy consumption can be in principle used as a part of any tandem integration. In this section, different CO_2_ capture methods that are explored in series with electrochemical conversion systems are emphatically discussed.

### 2.1 Direct Air Capture for CO_2_RR

**FIGURE 1:**
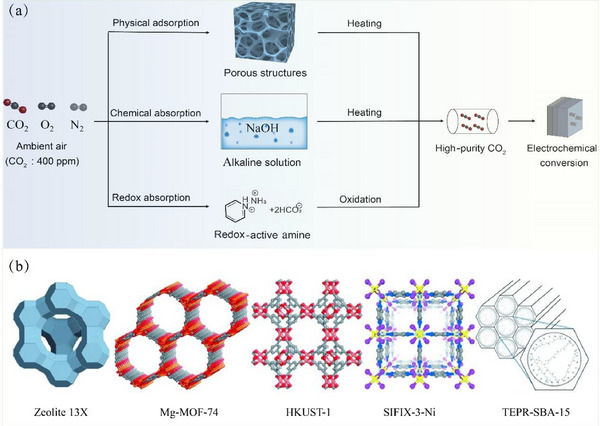
(a) Main routes for direct air capture. (b) Five adsorbents for physical (Zeolite 13X, Mg‐MOF‐74, HKUST‐1, and SIFIX‐3‐Ni) and chemical (TEPR‐SBA‐15) adsorption. Reproduced with permission [44]. Copyright 2015, WILEY‐VCH.

Direct air capture (DAC) is carried out by collecting CO_2_ from the atmosphere. Considering the low concentration of CO_2_ in air (approx. 400 ppm), it can be estimated that a large volume of air is required for the subsequent electrochemical conversion [35]. At present, three types of methods have been mainly used for the DAC, including physical adsorption, chemical absorption, and redox absorption (Figure 1a) [36, 37]. The consumption of energy in capturing CO_2_ by using physical adsorbents is lower than that by using chemical adsorbents. Nevertheless, chemical adsorbents possess a stronger capacity for capturing CO_2_, rendering chemical adsorbents more appealing in DAC. The redox capture technology can both capture and release CO_2_ and regenerate the capture solution via the redox reaction.

The physical adsorption method mainly utilizes absorbents with high surface areas, such as zeolite [38], metal–organic frameworks (MOFs) [39, 40], organic polymers [41], and porous carbon materials [42], among others. For example, the N‐doped porous carbon prepared by the pyrolysis of microporous polyindole exhibited a high CO_2_ adsorption capacity (1.42 mmol g^−1^) at room temperature (298K) due to its large Brunauer–Emmett–Teller (BET) surface area and high N content [43]. As for the chemical absorption, the concentrated alkaline solution is normally used due to its rapid reaction kinetics with CO_2_. For instance, sodium hydroxide (NaOH) can react with CO_2_ to form a carbonate (i.e., Na_2_CO_3_). Then, the calcium carbonate (CaCO_3_) is precipitated by reacting the Na_2_CO_3_ with Ca(OH)_2_, which is further calcined to release CO_2_ at high temperatures.

Both the physical adsorption and chemical absorption are facile, which however suffer from several challenges. For example, Kumar et al. [44] investigated the ability of four physical adsorbents and one chemical adsorbent to capture CO_2_, as shown in Figure 1b. It was found that all of the five adsorbents had strong CO_2_ adsorption capacity. However, the presence of water vapor would compete with CO_2_ for physical adsorption sites, resulting in a decreased performance for CO_2_ adsorption. Meanwhile, the main bottleneck for the chemical absorption is the high regeneration temperature required for the CO_2_‐releasing and adsorbent regeneration (e.g., >800°C for decomposing calcium carbonate), leading to intensive energy and adsorbent consumption [45].

**FIGURE 2:**
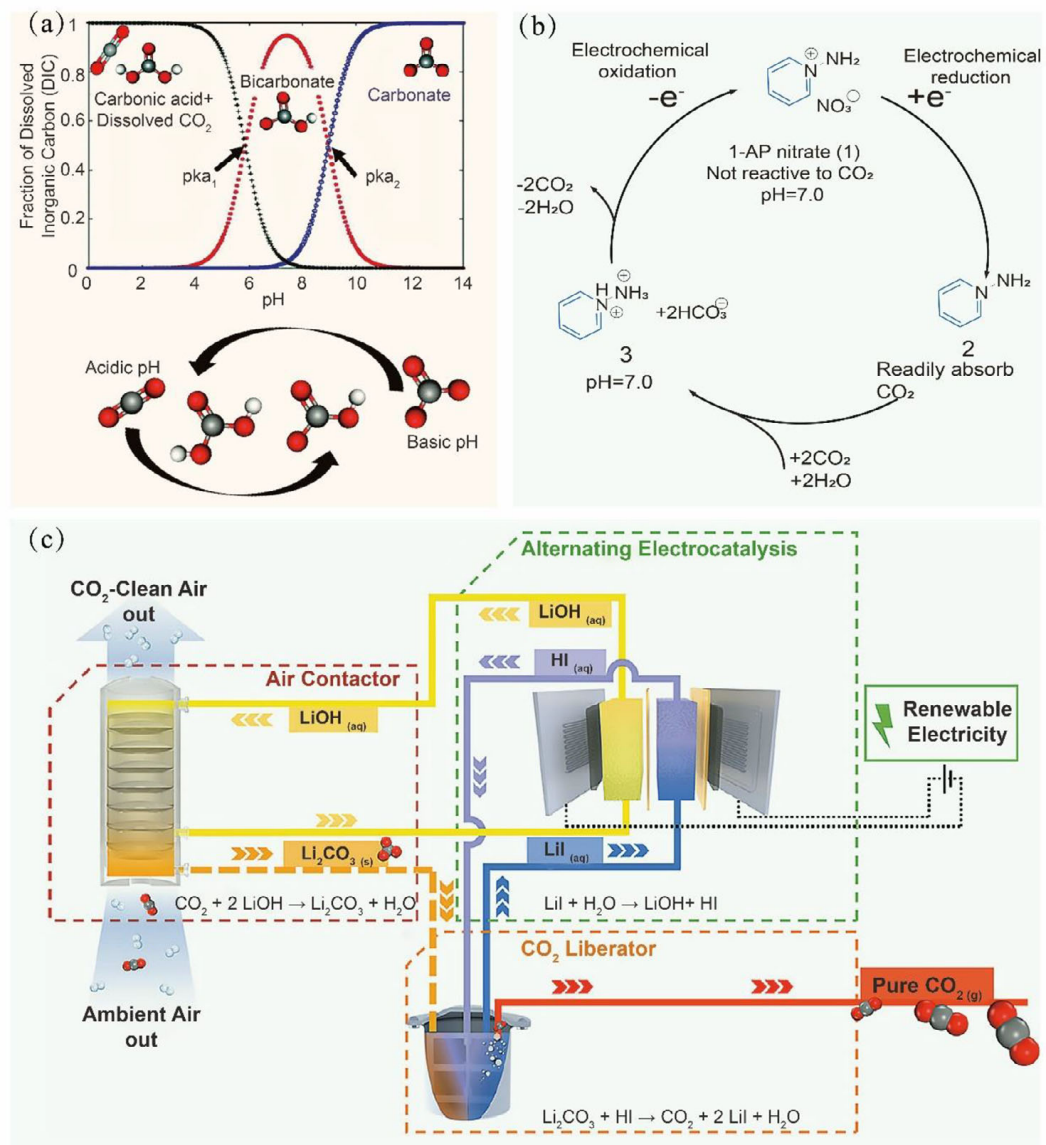
(a) Effect of pH on the CO_2_ equilibrium and schematic of the pH‐swing concept. Reproduced with permission [46]. Copyright 2021, The Royal Society of Chemistry. (b) Scheme for reversible electrochemical capture and release of CO_2_ using the 1‐AP nitrate for redox cycle. Reproduced with permission [48]. Copyright 2022, American Chemical Society. (c) An alternating electrocatalytic process for the capture liquid regeneration. Reproduced with permission [49]. Copyright 2023, Elsevier.

Recently, the concept of electrochemical capture has garnered attention with the merits of low energy consumption, flexibility, and sustainability. This method typically utilizes the redox trapping media or electrochemical manipulation of the solution pH to absorb or release CO_2_ [46, 47]. The pH‐swing‐assisted CO_2_ capture leverages the responsivity of CO_2_ thermodynamic equilibrium to pH changes (Figure 2a). A decrease in pH promotes the gaseous release of CO_2_ from the solution, whereas an increase precipitates CO_2_ reaction with OH^−^ to form bicarbonate or carbonate ions, thereby facilitating its capture. For instance, Seo et al. [48] reported the efficient and reversible electrochemical capture and release of CO_2_ by using the 1‐AP nitrate as an active amine absorber (Figure 2b). Initially, the 1‐AP nitrate was non‐reactive toward CO_2_. When it was electrochemically reduced to the electron‐rich 1‐APyl radical by single electron transfer, it could be stabilized by diamagnetic π‐dimer for capturing CO_2_ and producing two bicarbonate molecules per 1‐APyl radical. Then, the 1‐APyl‐bicarbonate solution was oxidized electrochemically to reproduce 1‐AP nitrate and release free CO_2_ to close the redox cycle. The resultant CO_2_ absorption efficiency was 12.4 mmol g^−1^, which is higher than that of the industrial capture process (8 mmol g^−1^) in the aqueous amine.

David et al. [49] also proposed an electrochemical technique by utilizing alternating electrocatalysis to regenerate the alkaline trap solution (Figure 2c). The LiOH and I_2_ were produced after the electrolysis in the LiI electrolyte. Subsequently, the formed I_2_ was electrochemically hydrogenated at the cathode and produced HI. Meanwhile, the LiOH absorbs CO_2_ to form Li_2_CO_3_, which then reacts with HI to release the pure CO_2_ and the LiI can be regenerated. This model facilitated the efficient regeneration of CO_2_ capture solutions by minimizing the membrane formation and loss during the regeneration of hydrogen halide and alkali hydroxide.

Although it is technically feasible to capture CO_2_ from the air, the release process will consume significant amounts of thermal energy and water, making DAC both energy‐intensive and not yet economically viable [36, 50, 51]. Accordingly, intensive research focus has been devoted to the search for alternative CO_2_ capture technologies. For example, Recker et al. [52] proposed an energy‐efficient strategy using ionic liquids (ILs), which contain a mixture of branched‐chain amino acids (valine, leucine, and isoleucine) that can repeatedly capture CO_2_ from the air, even in wet conditions. The molar uptake of CO_2_ per mole of IL was 0.5, approaching the theoretical maximum value for CO_2_ sequestration as carbamate salts.

Membrane separation technology is also emerging as an energy‐efficient strategy for DAC. This technology relies on the separation membranes with differential transmissivity and selectivity for various gas molecules, enabling the selective CO_2_ extraction from a gaseous mixture. Fujikawa et al. [55] proposed that membrane technology could complement the conventional adsorbent‐based DAC because the membrane separation technology can manage relatively smaller air volumes but is highly versatile in terms of installation locations owing to its compact footprint and scalable nature. Nonetheless, membrane separation technologies for CO_2_ capture are under‐researched and remain in the developmental stage, with no industrially viable membranes yet being developed specifically for DAC applications.

### 2.2 Marine Capture for CO_2_RR

The ocean represents the largest carbon sink in the world, absorbing approximately 40% of human‐induced CO_2_ emissions since the dawn of the Industrial Revolution. The higher CO_2_ concentration in seawater (∼100 mg/L) than that in the atmosphere (∼0.77 mg/L) provides more feasibility for capturing CO_2_ from the ocean, considering that the capture cost is highly sensitive to the CO_2_ concentration [56, 57].

**FIGURE 3:**
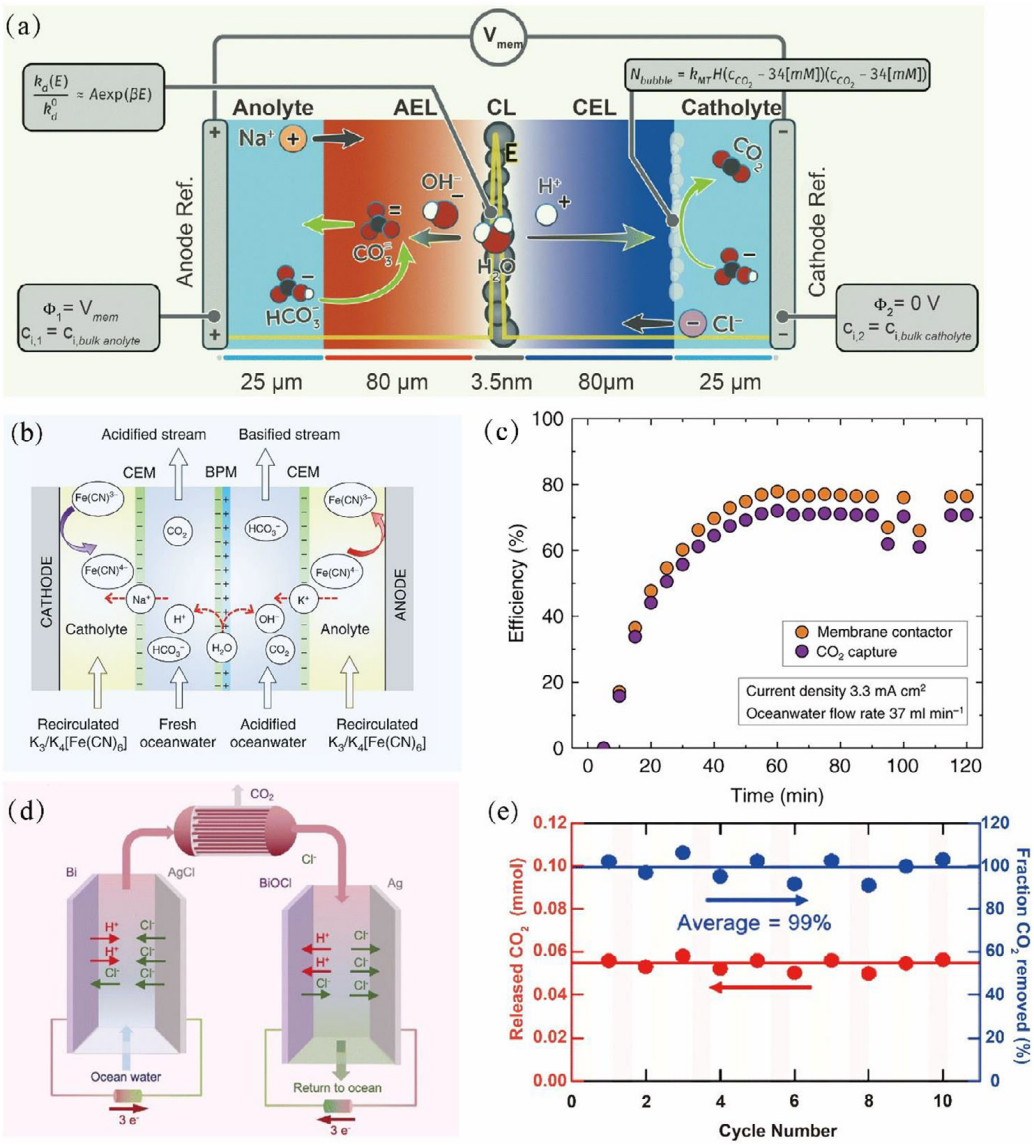
(a) Schematic diagram of CO_2_ capture in seawater by BPM electrodialysis system. Reproduced with permission [60]. Copyright 2023, The Royal Society of Chemistry. (b) Illustration for the working mechanism of an electrolysis unit using BPM capturing CO_2_ from the ocean. (c) Efficiencies of CO_2_ capture and membrane contactor. Reproduced with permission [64]. Copyright 2020, Springer Nature. (d) A device for CO_2_ removal from the seawater by a chloride‐mediated electrochemical pH swing system. (e) Molar amount of released CO_2_ (red, left) and a fraction of CO_2_ removed (blue, right). Reproduced with permission [66]. Copyright 2023, The Royal Society of Chemistry.

To date, the CO_2_ capture from seawater has predominantly relied on the electrolysis facilitated by the bipolar membranes (BPMs) [58, 59, 60]. The BPM is composed of a polymeric cation‐exchange layer (CEL) and an anion‐exchange layer (AEL). When subjected to the reverse bias, H_2_O is dissociated at the CEL/AEL interface to generate H^+^ and OH^−^, which will separately migrate to both sides driven by the electric field. The basic principle is that the generated H^+^ will acidify the seawater and precipitate CO_2_ from the seawater (Figure 3a). Subsequently, the acidified seawater passes through a liquid‐gas membrane contactor, where the dissolved CO_2_ is captured as gaseous CO_2_. Then, the acidified seawater is alkalized through the chamber to the right side of the BPM, and the pH value of the seawater is returned and discharged directly into the ocean [61, 62, 63]. Nevertheless, this electrodialysis process needs excessive energy consumption due to the occurrence of hydrogen and oxygen evolution reactions (HER and OER) at the cathode and anode, respectively. Recently, Digdaya et al. [64] proposed a directly coupled electrochemical system that substitutes the HER and OER with ferricyanide/ferrocyanide reduction couples present in the electrolyte (Figure 3b). This benefits an elevated CO_2_ capture efficiency (71%) with a lower electrochemical energy consumption of 155.4 kJ mol^−1^ CO_2_ (i.e., 155.4 kJ required for capturing 1 mol of CO_2_) compared to that of the BPM electrodialysis (300–1000 kJ mol^−1^ CO_2_; Figure 3c).

However, the incorporation of BPM with high prices and insufficient stability can inflate the overall installation costs and suffer from the risk of ferricyanide leakage into the ocean [65]. To circumvent these issues, Kim et al. [66] developed a CO_2_ capture device that eliminates the need for BPM and those types of chemicals. As portrayed in Figure 3d, the device comprises two serially connected Ag/Bi‐based electrochemical cells. In the left cell, the Bi electrode undergoes oxidation with H_2_O and Cl^−^ to form BiOCl, simultaneously releasing protons to acidify the seawater. Simultaneously, the AgCl electrode is reduced to Ag and releases Cl^−^. In the right cell, the inverse reactions occur by regenerating the electrodes while alkalinizing the seawater. This device thus achieves CO_2_ capture and electrode regeneration by a chloride‐mediated electrochemical swing system without the usage of BPM. Employing this device enables a CO_2_ removal rate from seawater as high as 99%, together with a releasing rate of 0.06 mmol CO_2_ per cycle (Figure 3e).

### 2.3 Flue Gas Capture for CO_2_RR

Direct capture of CO_2_ from the flue gas at industrial production sites, such as power plants and refineries, can be very beneficial for mitigating the release of CO_2_ into the atmosphere [67, 68, 69]. The relatively higher concentration of CO_2_ in flue gas (e.g., 20%–90%) compared to the air and ocean facilitates easier capture. Amine solutions, particularly monoethanolamide (MEA), are predominantly used in established industrial processes for capturing CO_2_ from flue gas due to their low cost and high chemical reactivity with CO_2_ [70, 71]. However, the regeneration of amine solutions is typically accomplished through heating, necessitating significant heat energy for CO_2_ desorption and purification [72]. For instance, the total cost of capturing a ton of CO_2_ is between $50–150 in the amine scrubbing process [73], with more than half of this cost attributed to the desorption and compression of CO_2_.

**FIGURE 4:**
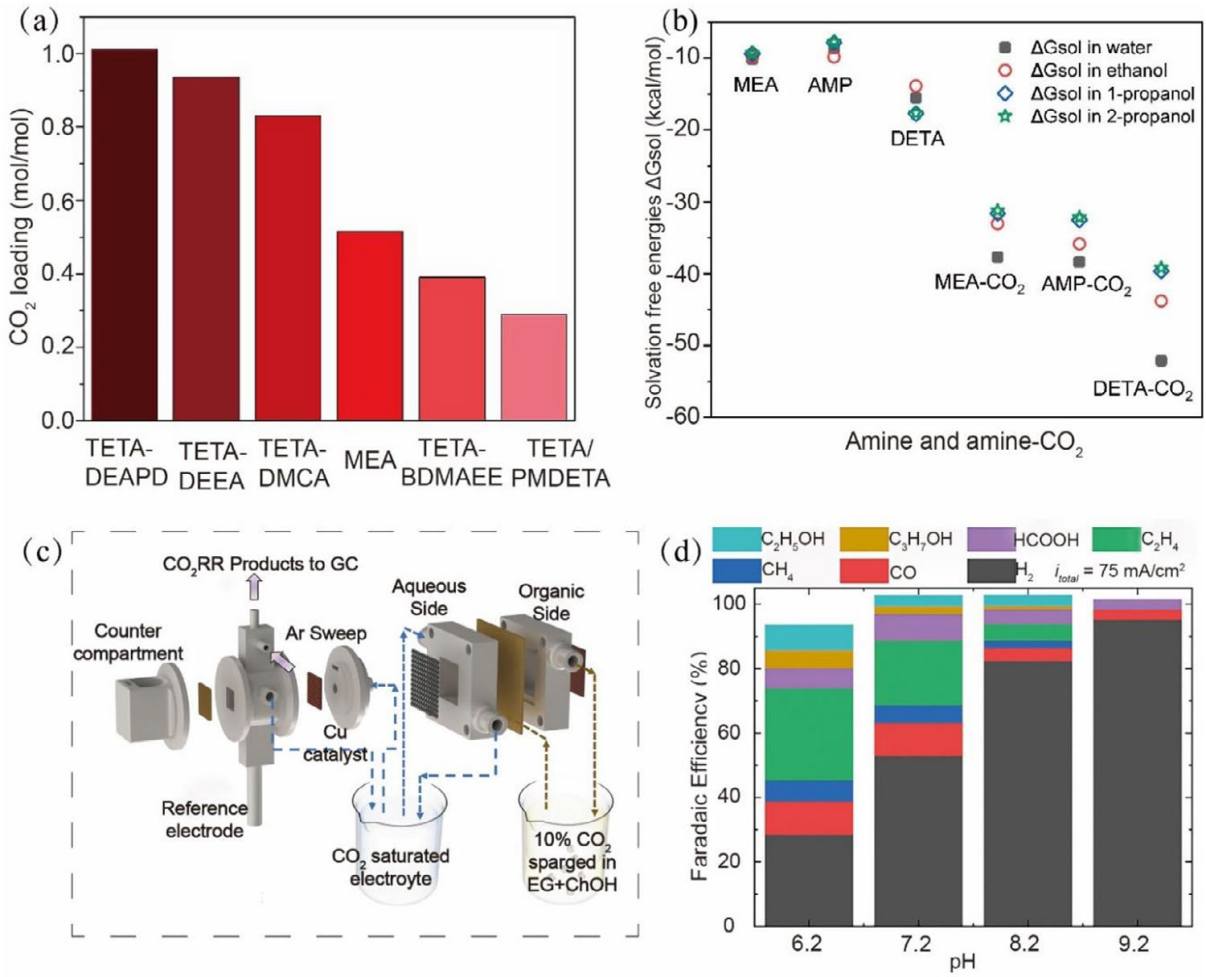
(a) CO_2_ absorption capacity of the tested five blends and MEA. Reproduced with permission [74]. Copyright 2018, American Chemical Society. (b) The solvation free energies. The greater the difference between the free energy of solvation in alcohol and in water, the more likely phase separation will occur. Reproduced with permission [75]. Copyright 2021, Elsevier. (c) Schematic of the integrated system with CO_2_ capture and electrochemical CO_2_RR. (d) The Faraday efficiency (FE) of products in different pH. Reproduced with permission [78]. Copyright 2022, The Royal Society of Chemistry.

To reduce the energy consumption of capturing CO_2_, Zhang et al. [74] developed a new phase‐change absorber consisting of a primary amine (triethylenetetramine, TETA) and a tertiary amine (DMCA), which showed a high CO_2_ capture capacity, fast absorption rate, and low energy loss. The underlying principle is that TETA, which contains two primary amine groups and two secondary amine groups, can form four carbamate esters through CO_2_ absorption. Meanwhile, DMCA transfers from the upper layer to the lower layer and makes TETA regenerated. Figure 4a displays the five mixtures and the ability of MEA to absorb CO_2_. It can be clearly seen that the CO_2_ capture capacity (1.0 mol mol^−1^) of TETA‐DEAPD as the biphasic solvent is twice that of MEA (0.5 mol mol^−1^). Therefore, this biphasic solvent can reduce the regeneration energy consumption by about 40% compared to the usage of MEA (the regeneration heat of the MEA is 3.7 GJ), making it a promising agent for capturing CO_2_ from flue gas.

The types of physical solvents also have a significant impact on the CO_2_ capture by the phase change absorbers. As a feasible physical solvent, alcohol can initiate and promote the reaction between amine and CO_2_. Li et al. [75] compared the CO_2_ adsorption capacity of five common amines (MEA, N‐methyldiethanolamine [MDEA], diethylenetriamine [DETA], TETA, 2‐amino‐2‐methyl‐1‐propanol [AMP]) and different combinations of the three alcohols (ethanol, 1‐propanol, 2‐propanol), as shown in Figure 4b. It revealed that the combination of DETA, alcohol, and water easily underwent phase separation after CO_2_ absorption due to the lower solubility of carbamate in alcohol. The optimized DETA/1‐propanol/H_2_O solution emerged as the superior adsorbent and further enhanced the adsorption capacity of CO_2_ to 1.53 mol mol^−1^ (i.e., each mol of DETA can capture 1.53 mol of CO_2_). Only the CO_2_‐enriched phase is sent to the stripper for adsorbent regeneration, significantly reducing the energy consumption of the desorption process.

Although phase‐change absorbers have greatly reduced the energy consumption of CO_2_ capture, the need for heating to regenerate the amine capture solution cannot be avoided. In addition, amine solutions have the characteristics of high volatility, toxicity, and corrosion, necessitating the development of more efficient and low‐cost CO_2_ capture technologies [12, 76]. Mezza et al. [77] reported a straightforward electrochemical system to capture CO_2_ from the gas mixtures and convert it to syngas. This system captures CO_2_ through the interconversion of NaHCO_3_ and NaOH. In the electrochemical flow cell equipped with a BPM‐based four‐compartment, the H^+^ (produced by the dissociation of water on the BPM) reacts with the bicarbonate, while the OH^−^ reacts with sodium ions to form NaOH to capture CO_2_ and produce NaHCO_3_.

Besides, Prajapati et al. [78] developed a system for the electrochemical capture of CO_2_ from flue gas according to the principle that HCO_3_^−^ reacts with H_2_O to form CO_2_ and CO_3_^2−^ (2 HCO_3_^−^ + H_2_O = CO_2_ + CO_3_^2−^; Figure 4c). In this system, the gaseous CO_2_ is captured as bicarbonate by an organic liquid. Under the influence of an electric field, the bicarbonate is transported through an anion exchange membrane (AEM) into an aqueous solution, where CO_2_ and CO_3_^2−^ are formed under water. By controlling the pH, a FE of 40% for producing the ethylene was achieved, and an efficient integration of CO_2_ capture and conversion was achieved (Figure 4d).

In the tandem systems, the gaseous CO_2_ serves as the direct feeding species for the conversion process. Therefore, a high‐performance CO_2_ capture system is particularly important, which can reduce energy consumption and capture purer CO_2_ as the feed of the conversion unit, thereby increasing the subsequent conversion efficiency. However, if the CO_2_ supplied from the capture systems exceeds the conversion system requirement, it could result in the carbonate crossover and wastage of CO_2_. Conversely, if the capture system performance is subpar, it may lead to insufficient CO_2_ being transferred to the electrolytic cell, thus hindering the achievement of optimal cell performance. As such, achieving a balance between the capture and conversion processes is crucial for efficient integration.

## 3 Direct Electrolysis of CO_2_ Capture Solutions

**FIGURE 5:**
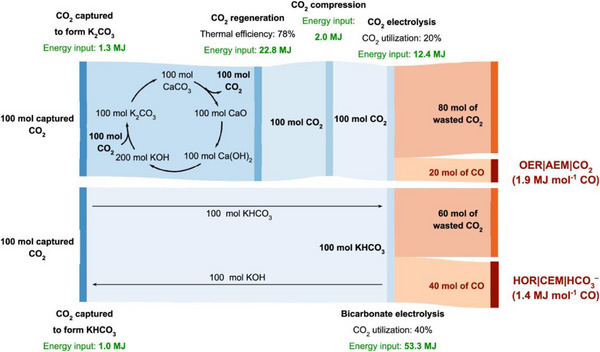
CO_2_ capture and utilization path and the corresponding energy input. Reproduced with permission [79]. Copyright 2022, American Chemical Society.

The tandem CO_2_ capture and electrochemical CO_2_ reduction processes discussed above still suffer from low carbon utilization, as they deliver high‐purity CO_2_ to the electrochemical reactor and a large percent of CO_2_ (over 80 vol.%) will be run away. A more effective strategy involves directly using the CO_2_ capture solution as the reaction species for CO_2_RR. Figure 5 shows the CO_2_ mass flow and energy input for the integrated and non‐integrated CO_2_ capture and conversion into CO as an example. Particularly, the integrated technology shows a higher CO_2_ utilization rate (40%) compared with that of the tandem methods (20%). Furthermore, it eliminates the need to release and pressurize CO_2_ from the capturing medium, thus significantly enhancing the energy efficiency by reducing the energy required from 1.9 to 1.4 MJ mol^−1^ CO. Currently, the commonly used CO_2_ capture solutions mainly include the alkaline hydroxide and amine solutions for the coupled strategies. As such, they are divided into two categories based on the different capture solutions: electrochemical conversion from the amine‐based capture fluid or the bicarbonate (or carbonate).

### 3.1 CO_2_ Conversion From Amine‐Based Capture Solutions

**FIGURE 6:**
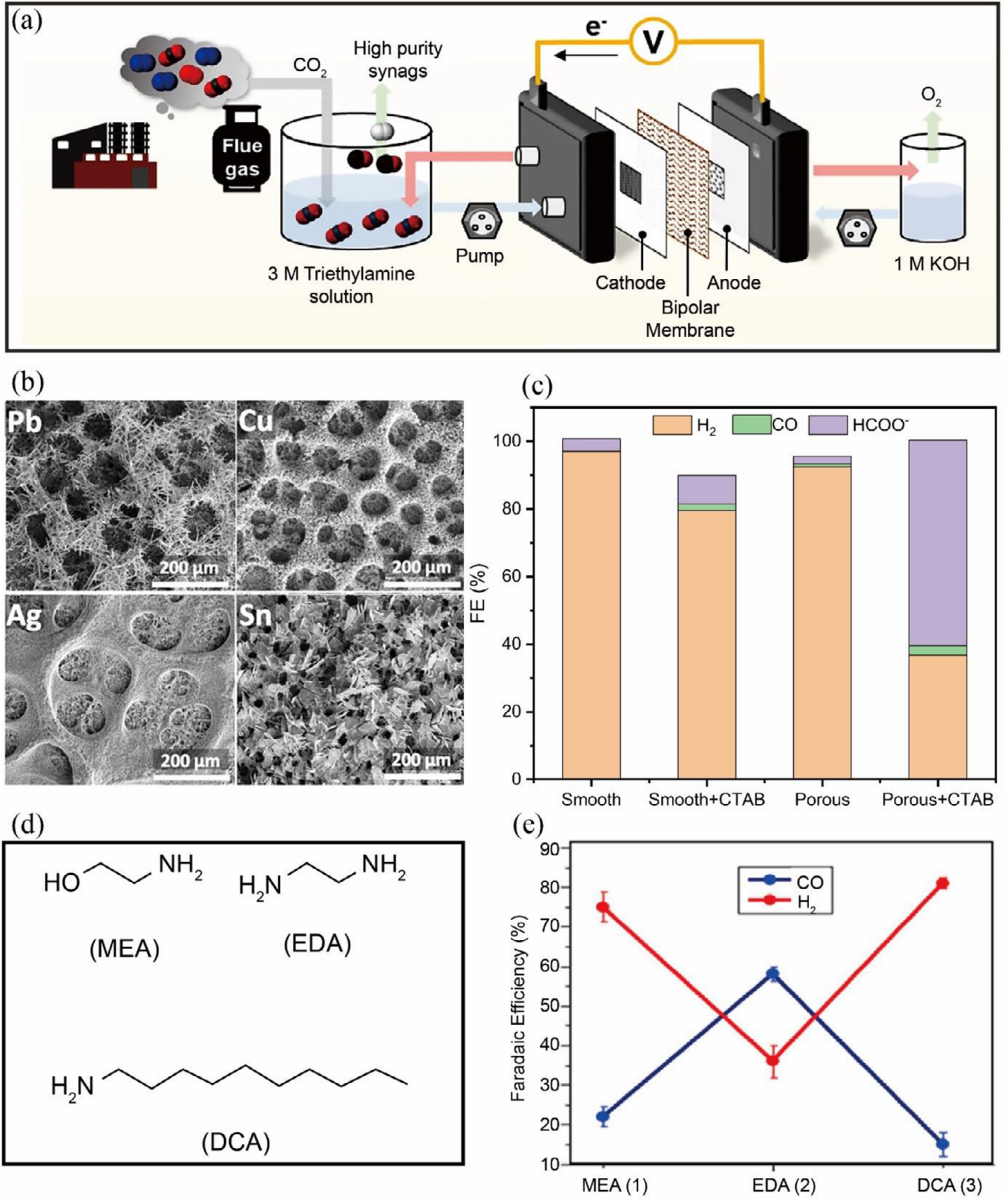
(a) Schematic diagram of a device for CO_2_RR using an amine capture solution. Reproduced with permission [72]. Copyright 2022, Springer Nature. (b) SEM images of various metals (Pb, Cu, Ag, Sn). Reproduced with permission [83]. Copyright 2022, WILEY‐VCH. (c) FE of CO and formate on Pb electrodes. (d) Molecular structure of several common amines. (e) FE of CO for various amines. Reproduced with permission [84]. Copyright 2020, American Chemical Society.

CO_2_ reduction from the amine‐based capture solution offers significant advantages. First, amine scrubbing is a well‐established technology for CO_2_ capture that has been widely utilized in industries for over 70 years [80]. Second, it bypasses the thermal regeneration of CO_2_ and the subsequent storage step, as illustrated in Figure 6a [72, 81]. In principle, amine‐based solutions can capture CO_2_ via the following reaction (Equation 1).

(1) $$2{\mathrm{RNH}}_{2}+{\mathrm{CO}}_{2}\to {\mathrm{RNHCOO}}^{-}+{\mathrm{RNH}}_{3}^{+}$$

Subsequently, the generated carbamate will be decomposed at temperatures of 100–120°C and in situ release CO_2_ gas around the electrodes. Then, CO_2_ is electrochemically converted into the value‐added products [82]. Chen et al. [83] reported on the direct electrochemical reduction of dissolved CO_2_ in MEA solution into CO and formate. Different catalysts were verified to be capable of yielding CO and formate (e.g., Pb, Cu, Ag, Sn; Figure 6b,c). In addition, it was demonstrated that the production of CO and formate originated from the CO_2_ in the MEA solution instead of the carbamate, offering valuable insights for future research on the electrolysis of amine‐based capture solutions.

Direct electrolysis of amine‐based capture solution however suffers from insufficient current density and FE compared to the directly gaseous CO_2_ fed electrolyzers. The primary cause for this gap is due to the small amount of dissolved CO_2_ and thus insufficient CO_2_ adsorption onto the catalyst surface. Kraatz et al. [84] carried out a systematic evaluation of several commonly used primary amines including MEA, ethylenediamine (EDA), and decylamine (DCA) (Figure 6d). DEA was found to have the highest efficiency due to its two amino groups with dual capture sites, which enables the capture of more CO_2_. As a result, both the FE and current density of CO were significantly enhanced (the FE of 58% and current density of 18.4 mA cm^−2^), greatly surpassing that achieved with the standard MEA solution (the FE of 22% and current density of 14.8 mA cm^−2^; Figure 6e).

Pérez‐Gallent et al. [85] proposed an intriguing method using a blend of chemical and physical CO_2_‐absorbing solvents as the capture fluid. A mixed solution of AMP and propylene carbonate (PC) simultaneously worked as a CO_2_ capture solution and the electrolyte, increasing the concentration of CO_2_ in the solution, achieving a high FE of 45% and current density of 10 mA cm^−2^ for producing formate. The concentration of the AMP was also found to play a key role in the product selectivity, verified by a 50% increase of the FE_formate_ when the solvent concentration was improved from 0 to 2 M.

**FIGURE 7:**
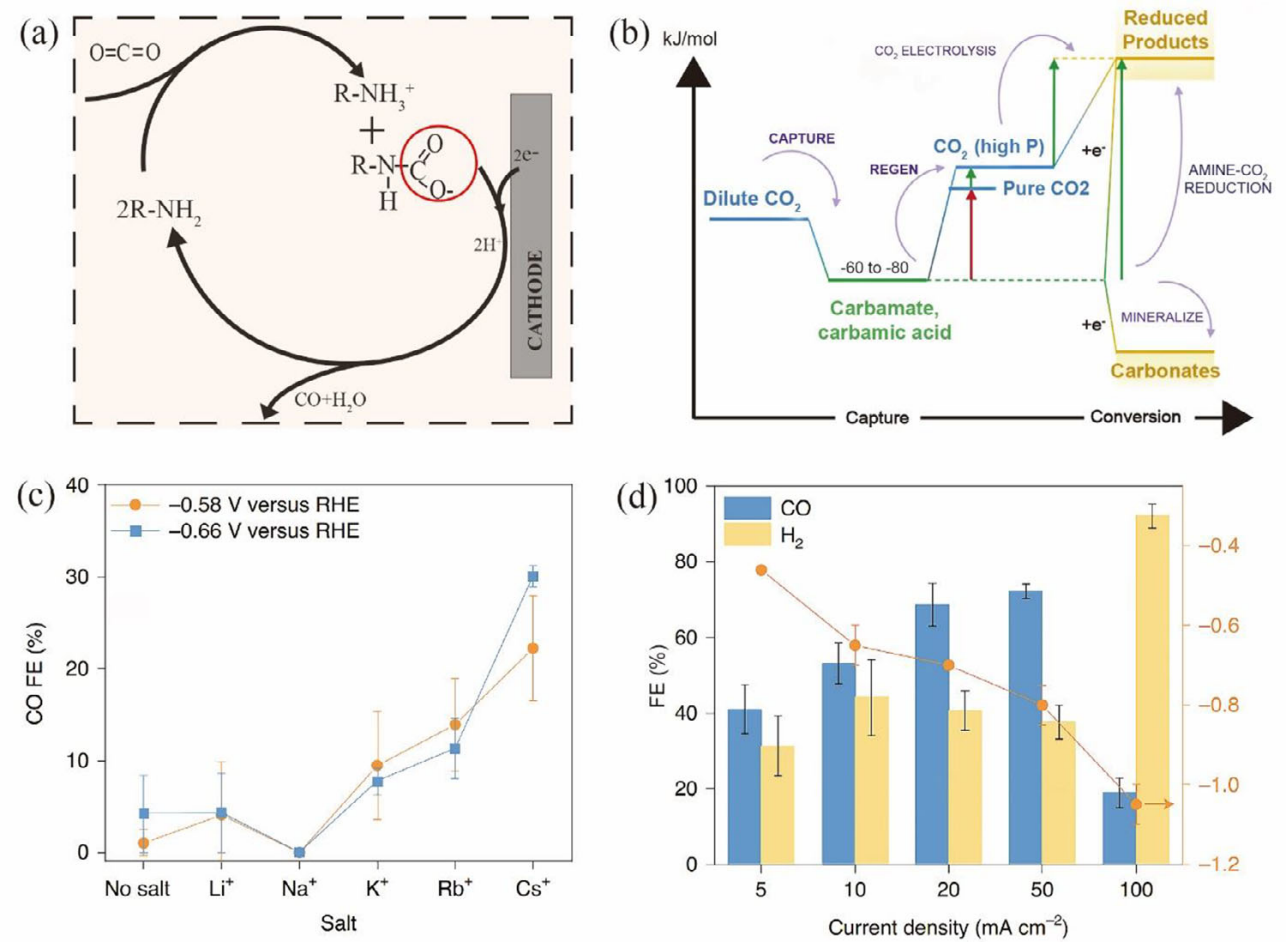
(a) Electrolytic process of amine‐CO_2_ adducts. (b) Free energy landscape of a process using amines. The red arrows represent thermal energy requirements; the green arrows represent renewable electricity. Reproduced with permission [86]. Copyright 2022, Elsevier. (c) The FE for different alkali cation salt solutions in the MEA electrolyte. (d) Product distribution of MEA‐CO_2_ conversion to H_2_ and CO in a flow‐cell system at different current densities. Reproduced with permission [89]. Copyright 2020, Springer Nature.

Compared to the in situ generated CO_2_ from the carbamate, the direct electrolysis of amine‐CO_2_ adducts (i.e., the carbamate) at the cathode has recently gained considerable interest. After CO_2_ is captured as a carbamate, it changes from a stable linear molecular structure to an activated nonlinear structure. The kinetic barrier of CO_2_ conversion can be decreased and the selectivity of products can be increased (Figure 7a) [86]. In the electrolysis process, the thermal energy requirement of CO_2_ regeneration (red arrow) is completely replaced by renewable electricity (green arrow), which avoids the thermal regeneration step of CO_2_, thus simplifying the production process and saving costs (Figure 7b). Khurram et al. [87, 88] studied the role of basic cations in the electrolysis of amine‐CO_2_ adducts. It showed that the presence of cations considerably influences the thermochemistry of amine‐CO_2_ through electrostatic interactions. In the absence of alkali metal cation (e.g., Li^+^, Na^+^, K^+^), carbamate could not be electrochemically reduced. However, as the size of the cation was decreased, the interaction between the cation and carbamate was strengthened, leading to the formation of more carbamate and a consequent increase in the reduction current. Therefore, the electrochemical reaction rate of amine‐CO_2_ adducts can be significantly enhanced by tuning the cation–solvent–amine interaction.

Although the direct electrolysis of amine‐CO_2_ adducts is feasible, this method still faces challenges in terms of low current density and low CO_2_ conversion efficiency due to the limited concentration. Lee et al. [89] optimized the electrochemical double layer by using alkali metal cations to migrate the amine‐CO_2_ adduct to the reaction site. It was demonstrated that cations adsorbed on the electrode surface promoted the transfer of electrons. When alkali cations were introduced to the electrolyte, they facilitated the electron transfer to the carbamate, bringing the amine‐CO_2_ adduct closer to the heterogeneous reaction site. This improved the selectivity of converting CO_2_ to CO, enabling direct electrolysis of the amine‐CO_2_ adduct at 50 mA cm^−2^ with a FE_CO_ of 72%, as shown in Figure 7c,d.

Despite the basic feasibility of direct electroreduction of amine‐CO_2_ adducts and its capability of yielding products such as CO and formate, with high selectivity, the research is still in its early stages and there are many aspects that are yet to be fully explored. Factors such as the type of electrolyte salt and solvent, as well as the influences of mass transfer and electron transfer, make the electrolysis system very complex [86]. A deeper understanding of these factors will facilitate a more precise evaluation of the electrolysis system and aid in the design of the electrochemical reactor.

### 3.2 CO_2_ Conversion From Bicarbonate (or Carbonate)

Utilizing a strong base like KOH as a capture liquid enables CO_2_ to be captured in the form of bicarbonate or carbonate, which can be easily transported into the electrochemical reactor [90]. Direct electrolysis of bicarbonate is intriguing as it achieves higher reduction currents due to the higher ionic conductivity of bicarbonate compared to the amine‐based solutions. Moreover, it can bypass the energy‐intensive steps such as CO_2_ release from the capture medium and compression process, which reduces energy consumption [91]. For example, Yue et al. [92] compared the energy requirements for direct electrolysis of bicarbonate (carbonate) and electrolysis of gaseous CO_2_ to produce CO or formate. The results show that the energy requirement of direct electrolysis of carbonate (21.8 GJ) is much less than that of electrolysis of gaseous CO_2_ (34.2 GJ), which mainly originates from avoiding CO_2_ desorption, compression, and product purification.

Debates remain as to whether the bicarbonate acts directly as a reaction species or as a carbon donor in the electrolysis process [93, 94]. An accurate understanding of this mechanism can help further optimize the electrochemical systems for CO_2_ reduction. Dunwell et al. [95] found that the bicarbonate can be used as the carbon source in addition to proton donor and pH buffer because most of the CO_2_ in the electrolyte comes from the balance with bicarbonate, rather than the diffusion of CO_2_. When the bicarbonate concentration is higher than 1 M, it will provide protons to the electrolytic system and lead to the occurrence of competing HER [96]. However, Shen et al. [97] theoretically and experimentally revealed that the CO_2_ involved in the reaction when electrolyzing CO_2_ capture liquid came from the decomposition of the CO_2_ capture agent (e.g., bicarbonate and amine‐based capture) and the uncombined dissolved CO_2_. Only at a high negative potential, in the case of amine‐based capture, can carbamates be electrolyzed directly. Sanchez et al. [98] employed the cationic surfactants (cetalkonium chloride (CKC)) to cover the electrode surface to suppress the proton provided by bicarbonate. This allowed nonpolar CO_2_ molecules to access the electrode surface while inhibiting the bicarbonate and water from promoting the HER, thereby achieving a good FE_formate_ of about 70%. Therefore, strategies toward inhibiting the competing HER are very important to achieve the high selectivity and high current density of reductive products when the bicarbonate is directly electrolyzed.

#### 3.2.1 Electrolytic Cell System Based on BPM

Improving the carbon donor capacity of bicarbonate and increasing the CO_2_ concentration near the electrode surface has become the key focus to enhance the FE and current density of the reductive product. Currently, the main strategy is to employ the electrolysis of bicarbonate in BPM‐based flow cells.

**FIGURE 8:**
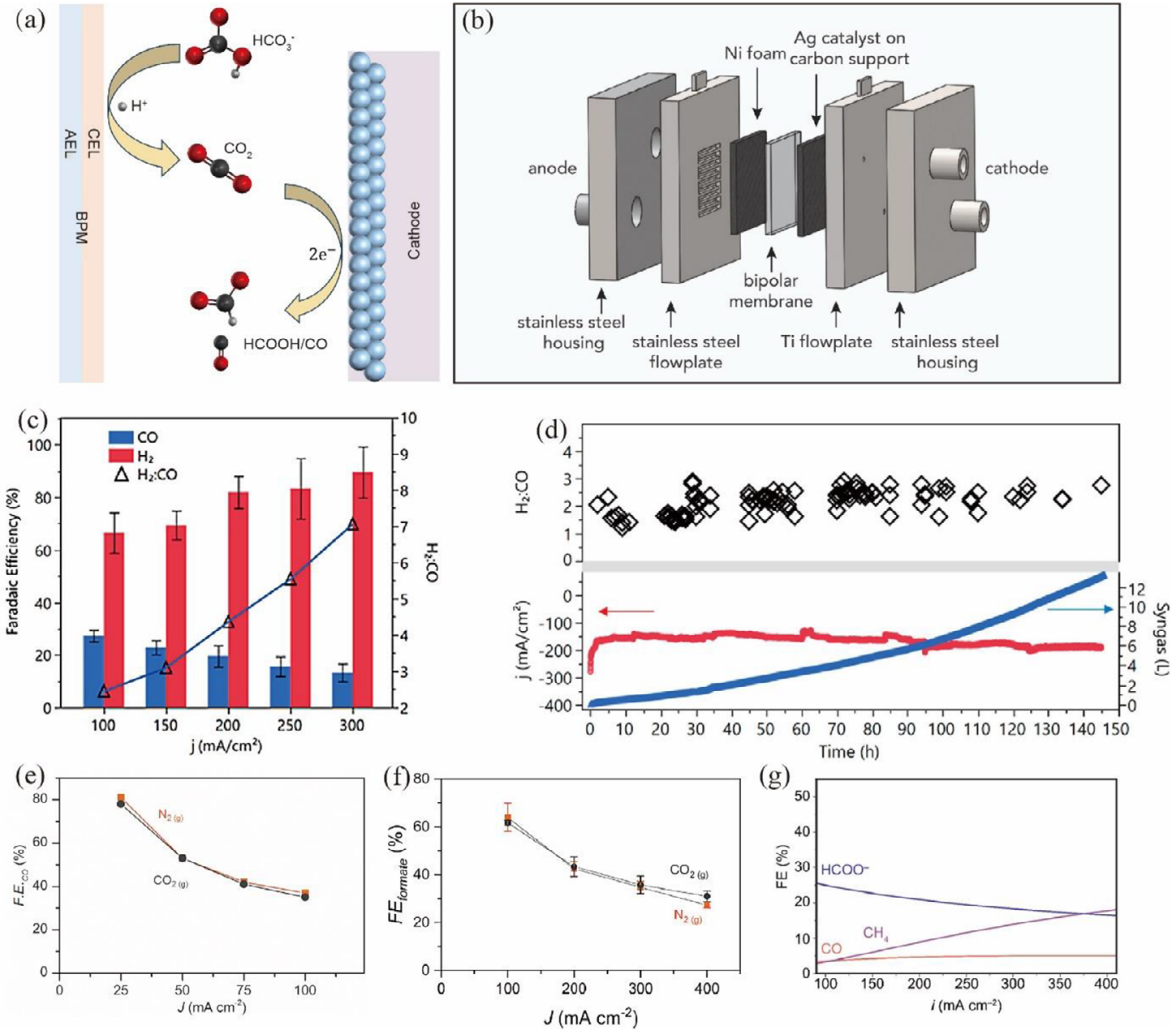
(a) Schematic diagram of in situ CO_2_ generation by BPM. (b) Structure of electrolytic cell for electrolysis of bicarbonate. Reproduced with permission [107]. Copyright 2020, American Chemical Society. (c) FE_H2_, FE_CO_, and their molar ratio. (d) Stability evaluation of carbonate electrolyzer using Ag nanoparticles as catalyst. Reproduced with permission [99]. Copyright 2019, American Chemical Society. (e) FE_CO_ by using Ag nanoparticles. Reproduced with permission [100]. Copyright 2019, Elsevier. (f) FE_formate_ by using Bi nanoparticles as catalyst. Reproduced with permission [101]. Copyright 2022, American Chemical Society. (g) FE_CH4_ by using a porous copper electrode. Reproduced with permission [109]. Copyright 2022, American Chemical Society.

In the BPM‐based system (Figure 8a,b), the water first dissociates into H^+^ and OH^−^ on the BPM (Equation 2). The H^+^ and bicarbonate then react on the cathode to generate in situ CO_2_ and water (Equation 3). The generated CO_2_ then migrates to the electrode surface as a reaction substrate (Equation 4). Meanwhile, the protons generated at the BPM inhibit the proton donor capacity of the bicarbonate.

(2) $$H_{2}O\to H^{+}+{\mathrm{OH}}^{-}$$

(3) $$H^{+}+{\mathrm{HCO}}_{3}^{-}\to {\mathrm{CO}}_{2}+H_{2}O$$

(4) $${\mathrm{CO}}_{2}+H_{2}O+ne^{-}\to \text{product +}\;{\mathrm{OH}}^{-}$$

Li et al. [99] used a BPM to generate protons in situ to facilitate CO_2_ release from bicarbonate at the membrane‐catalyst interface. Ag nanoparticles were used as the catalyst, which achieved the production of syngas at a ratio of 3:1 (H_2_: CO), as shown in Figure 8c. CO_2_ was not detected at the output and achieved 100% carbon utilization in the whole system. In addition, it remained stable after 145 h of continuous operation at a current density of 150 mA cm^−2^ (Figure 8d). In the same year, Berlinguette et al. [100, 101] also built an electrochemical flow reactor with a BPM and used the porous carbon scaffolds coated with Ag nanoparticles as cathodes to increase the electrochemically active areas. It was demonstrated with FE of 81% for CO production at 25 mA cm^−^^2^ with a 3.0 M KHCO_3_ electrolyte (Figure 8e). This result can be comparable to that of electrolysis of pure gaseous CO_2_. In addition, an FE_formate_ of 64% was achieved using a Bi catalyst at a current density of 108 mA cm^−2^, as shown in Figure 8f.

Despite these advancements, further improvement is still expected in terms of the electrolysis of bicarbonate in the flow cell. Compared with the regular electrolyzers fed with high‐purity CO_2_, the larger overpotentials and thicker BPM result in the increased ohmic loss of the electrolyzer and thus decreased energy efficiency [102, 103]. Also, the protons generated by the BPM may still promote the HER due to the production of protons. In addition, the use of BPM adds to the overall cost of the electrolyzer. Therefore, further optimization of the reactor structure and components, such as BPM, gas diffusion electrodes (GDEs), and electrocatalysts, is still highly necessary [104].

#### 3.2.2 Optimization of BPM‐Based Electrolyzers

At present, the maximum energy efficiency (EE) of direct electrolysis of bicarbonate is only 27% (comparable to the EE of electrolysis of gaseous CO_2_). One of the biggest reasons for lower EE is an increase in battery voltage due to the use of BPM [105]. Therefore, it is still necessary to optimize the individual components of the electrolysis system.

**FIGURE 9:**
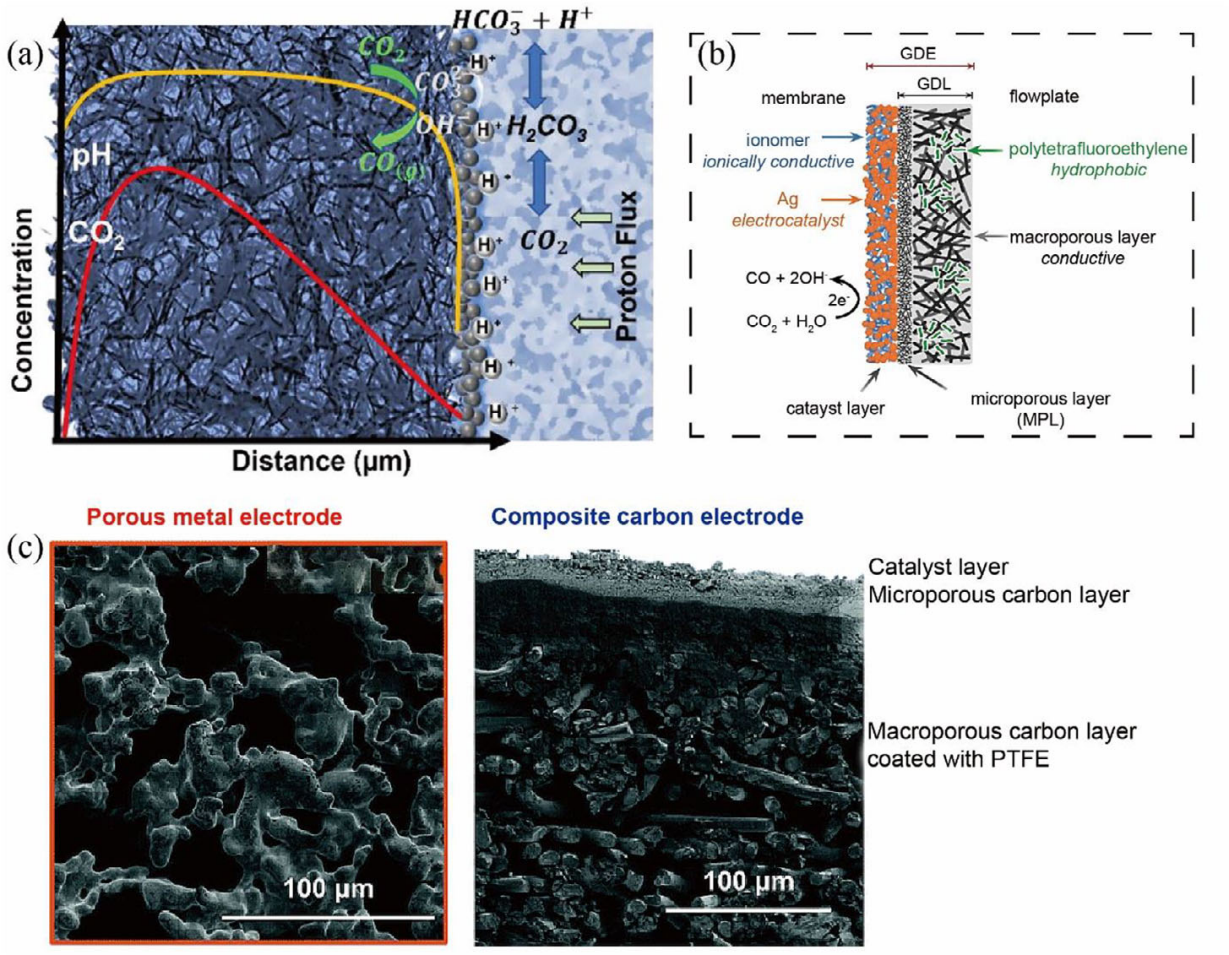
(a) Schematic diagram of bicarbonate diffusion model. Reproduced with permission [106]. Copyright 2022, American Chemical Society. (b) Cross‐sectional schematic diagram of a silver GDE. Reproduced with permission [107]. Copyright 2020, American Chemical Society. (c) scanning electron microscopy (SEM) images of the free‐standing porous metal electrode and the composite carbon electrode. Reproduced with permission [108]. Copyright 2022, The Royal Society of Chemistry.

Kas et al. [106] reported the model related to the mass transfer of bicarbonate and CO_2_ in GDE, to gain insights into the local concentration of species within a porous electrode (Figure 9a). It showed that a large amount of CO_2_ was produced in the porous electrode during the electrochemical reduction process. The CO_2_ source came from the decomposition of bicarbonate in the liquid phase, rather than the supply of gaseous CO_2_. Therefore, in the electrolytic bicarbonate system, it is not necessary to use the gas diffusion layer in the GDE structure to transport gaseous CO_2_. However, since the protons are neutralized by OH^−^, a byproduct of the CO_2_ reduction reaction, the CO_2_ concentration in the electrode stabilized in a certain concentration range, which limited the increase in the current density of the product. There was a large amount of carbonic acid (0.1–1 mM) and a low pH near the membrane, which was locally favorable for HER. It is necessary to study the concentration distribution of CO_2_ supply in bicarbonate systems, which is important for rationally optimizing the structure of GDEs and enhancing the in situ CO_2_ generation.

Based on the previous studies, Berlinguette et al. [107, 108] further modified the Ag GDE architecture by removing the commonly used hydrophobic components (e.g., PTFE), to promote the distribution of bicarbonate and increase the local CO_2_ concentration. In addition, the load and coverage of the catalyst were also optimized, which was also demonstrated to be critical for increasing the FE_CO_. As a result, it achieved the conversion of the captured solution to CO at high current densities (>100 mA cm^−2^; Figure 9b). By eliminating the PTFE and microporous layer in GDE and optimizing the catalyst coverage (95%) and load (1.3 mg cm^−2^), a higher FE and product current density were achieved (FE_CO_ of 82% at the current densities of 100 mA cm^−2^). Later, they further reported (Figure 9c) the design of a freestanding porous Ag electrode instead of using the carbon‐based GDE, which showed the merits of superior stability, easier assembly, and reusability, with the achievement of a high FE_CO_ of 95% at the current density of 100 mA cm^−2^ and 0.4 MPa. Then, Lees et al. [109] achieved a methane yield of 34% by adding a surfactant to the porous Cu electrode, which inhibited CuO formation and HER during electrolysis and promoted the CO_2_RR by creating a highly alkaline environment near the catalyst (Figure 8g).

#### 3.2.3 BPM‐Free Strategies

**FIGURE 10:**
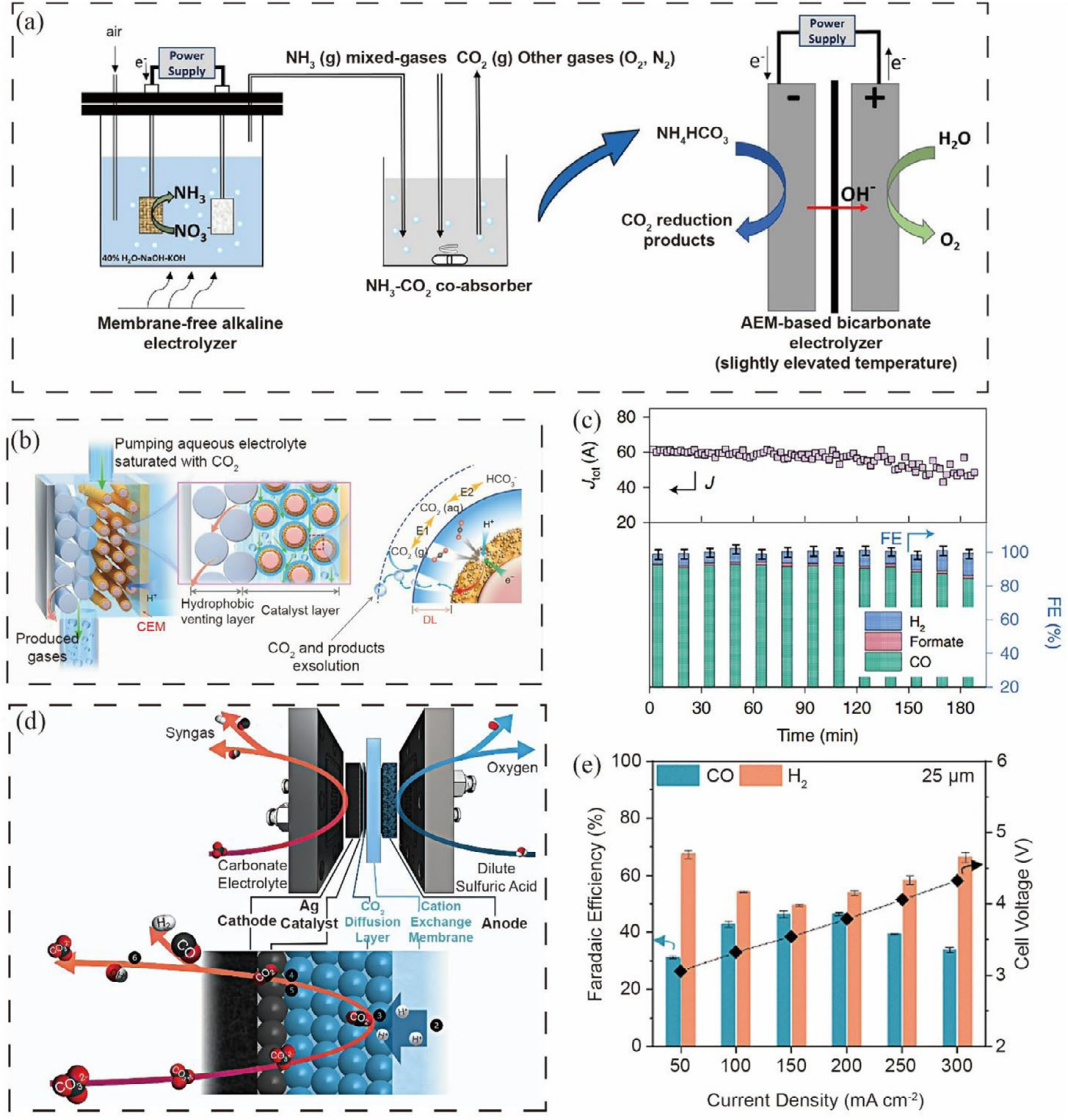
(a) Schematic diagram of the combination of ammonium bicarbonate electrolysis and nitrate electroreduction. Reproduced with permission [110]. Copyright 2022, Springer Nature. (b) In situ dissolution of CO_2_ by changing the pressure drop. (c) The stability of the porous electrode in 180 h. Reproduced with permission [111]. Copyright 2022, American Chemical Society. (d) CO_2_ diffusion layer was added between the catalyst and the ion exchange membrane. (e) Adsorption layer strategy was used to produce FE of syngas. Reproduced with permission [113]. Copyright 2022, The Royal Society of Chemistry.

Although the BPMs promote in situ CO_2_ generation, the high cost and thickness compared to the other ion‐exchange membranes significantly increase the ohmic loss in the system. Hence, alternatives to BPM‐based electrolyzers are expected and proposed. One potential solution involves utilizing the dissolution equilibrium of CO_2_ in solution to release CO_2_, represented by these equations (CO_2_(l) ⇌ CO_2_(g) or ${\mathrm{HCO}}_{3}^{-}⇌$ CO_2_(g)). Liu et al. [110] reported the superior electrolysis performance using ammonium bicarbonate as a feedstock compared to the state‐of‐the‐art KHCO_3_ electrolysis. The concentration of CO_2_ was twice upon the thermal decomposition of ammonium bicarbonate. Combining the ammonium bicarbonate electrolysis with nitrate electroreduction allowed for CO_2_ capture using the produced ammonia, and the BPM can be replaced by an AEM, resulting in FE_formate_ of more than 90% and 99.8% utilization of CO_2_ capture agent (Figure 10a). Compared with BPM‐based cells, the use of AEM reduced the battery voltage by 1.3 V and thus reduced the cost of the electrolysis system.

Wen et al. [111] proposed a novel electrolyzer structure by reducing the local pressure to promote CO_2_ release from the solution (Figure 10b). When the solution flows through a smaller channel within the porous electrode, the decrease in pressure reduces the solubility of CO_2_, leading to the generation of CO_2_ from the CO_2_‐saturated potassium bicarbonate solution. This electrolyzer achieved a high FE_CO_ of 92% at a current density of 1.75 A cm^−2^, which could stably run for 180 h (Figure 10c).

Using an acidic electrolyte as the anolyte and a cation exchange membrane to provide protons to the cathode can also regenerate CO_2_ in situ from bicarbonate. However, excess protons will lower the pH around the cathode, leading to enhanced HER and thus reduced product selectivity. Lees et al. [112] investigated the mechanisms of in situ CO_2_ generation and reduction, indicating that a low CO_2_ concentration in the catalyst layer impedes CO_2_ reduction at high current density. It was concluded that the acidic film layer to promote the formation of CO_2_ in situ and the alkaline catalyst layer to inhibit the HER are the key factors to promote the rapid formation of CO. Therefore, adjusting the thickness of the membrane and catalytic layer, designing the electrode structure, or covering a protective layer on the surface of the catalytic layer can limit the diffusion of H^+^ to the surface of the catalyst, which can form a highly alkaline microenvironment near the catalytic layer and promote CO_2_RR.

Xiao et al. [113] reported the electrolysis of bicarbonate under acidic conditions by adding a CO_2_ diffusion layer between the cathode catalytic layer and the cation exchange membrane (Figure 10d). This diffusion layer consisted of hydrophilic ionomers combined with TiO_2_ nanoparticles. With the presence of the diffusion layer, a pH gradient was created on the cathode side of the electrolyzer, maintaining a high pH near the catalyst surface and thus enabling a high FE_CO_. At a current density of 200 mA cm^−2^, the ratio of H_2_:CO reached 1.16, as shown in Figure 10e. This novel design could potentially inspire the design of efficient and cost‐effective electrolysis systems.

## 4 Electrocatalysts for Local CO_2_ Enrichment and Conversion

**FIGURE 11:**
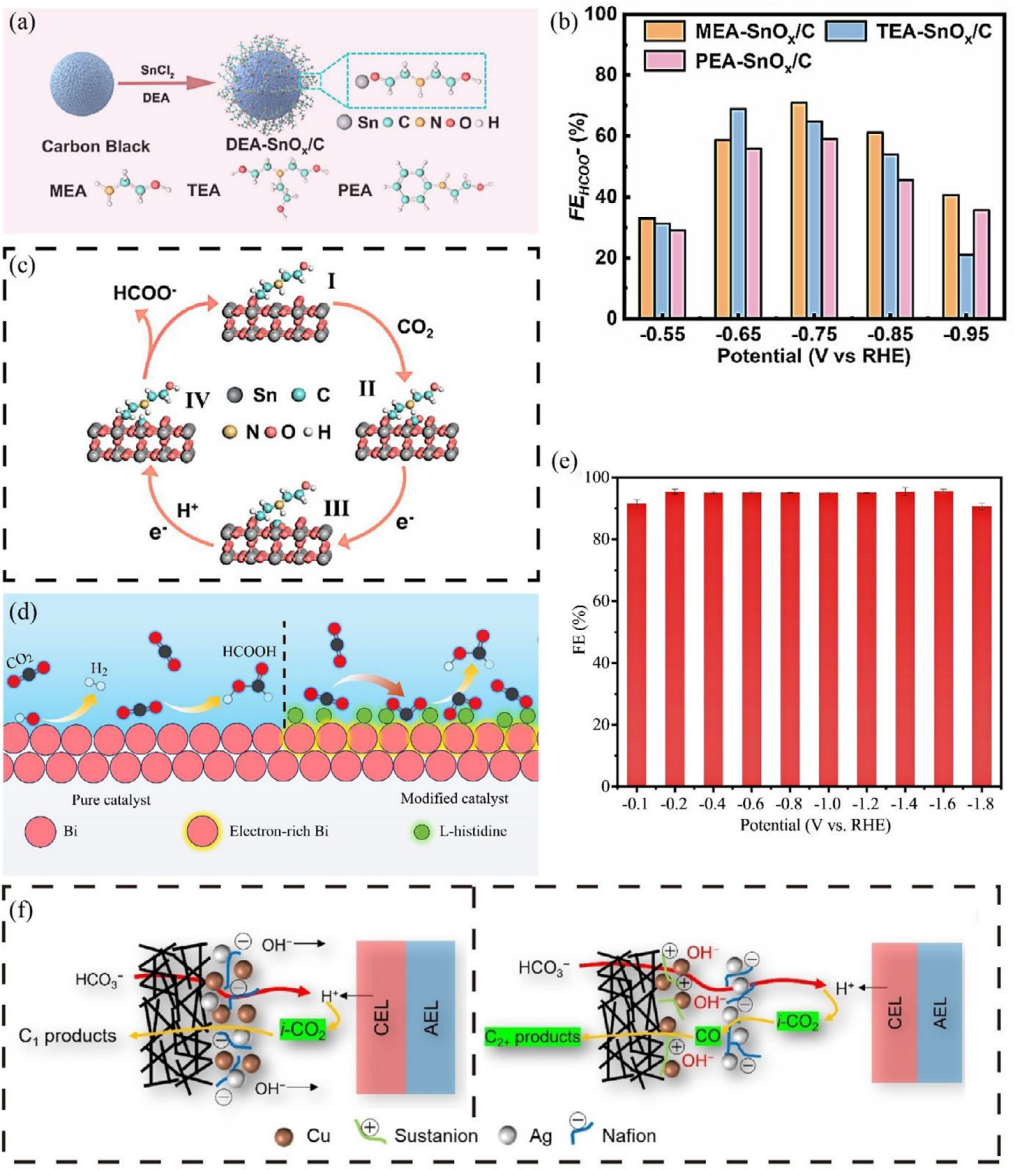
(a) Amine‐functionalized tin dioxide nanoparticle catalyst. (b) The functionalized catalyst produces FE of formate. (c) Schematic illustration of the proposed mechanism for formate formation over DEA−SnOx/C catalyst. Reproduced with permission [118]. Copyright 2023, WILEY‐VCH. (d) Bi‐based catalysts modified with L‐histidine (e) The FE_formate_ by using Bi‐based catalysts modified with L‐histidine. Reproduced with permission [119]. Copyright 2022, American Chemical Society. (f) Schematic illustration of the microstructure of CuAg Naf (left) and Cu Sus/Ag Naf (right). Reproduced with permission [132]. Copyright 2022, WILEY‐VCH.

Beyond the tandem systems and direct electrolysis of CO_2_ capture solutions, another promising strategy involves the development of efficient catalysts with excellent ability of local CO_2_ enrichment [114, 115]. The high‐performance catalyst can not only enrich CO_2_ locally but also improve the current density and stability [116, 117]. Cheng et al. [118] managed to functionalize the catalyst by attaching organic functional groups to the surface of tin oxide. This catalyst enables the CO_2_ enrichment and conversion to formate in a simulated flue gas atmosphere (Figure 11a–c). The experimental results showed that the diethanolamine‐modified tin oxide catalyst under a simulated flue gas inlet atmosphere achieved a partial current density of 6.7 mA cm^−2^ of formate and a maximum FE of 84.2%. The amino group on the surface can not only enrich the local CO_2_ but also inhibit the reduction of oxygen, demonstrating the high oxygen tolerance of catalysts. Li et al. [119] enhanced the adsorption capacity of CO_2_ by incorporating L‐histidine with bismuth‐based catalysts (Figure 11d). The results showed that the catalyst had excellent CO_2_ chemisorption and electron‐rich properties compared to traditional DEA‐ and TETA‐modified Bi‐based catalysts, contributing to the achievement of more than 90% FE_formate_ in an ultrawide potential window (0.1–1.8 V), as shown in Figure 11e.

Furthermore, nitrogen doping in the carbon‐based catalysts can provide the basic sites, which is beneficial for improving the CO_2_ adsorption capacity of the electrocatalyst [120, 121]. Li and Yan et al. [122, 123] demonstrated that Zn/Ni‐ZIF‐8‐1000 with high CO_2_ adsorption capability (4.25 mmol g^−1^) exhibited outstanding CO_2_RR performance with FE_CO_ of more than 92%. Their results suggest that those catalysts containing N basic groups could serve as a bifunctional catalyst for coupled CO_2_ capture and conversion in the future.

The direct electroreduction of CO_2_ to C_2+_ products is of great interest due to its higher market size and value [121, 122, 123, 124, 125, 126]. Although the coupled CO_2_ capture and conversion technology has made great progress in producing C_1_ products, the conversion of bicarbonate into C_2+_ products has been limitedly studied, and the reported FE_C2+_ is still very low. For example, Li et al. [99] reported that the electrolysis of bicarbonate only achieved a FE_C2+_ of 17%, which is far from the requirements for practical applications. The difficulty in achieving C_2+_ products from CO_2_RR can be mainly ascribed to the insufficient CO_2_ supply and thus limited C‐C coupling steps.

At present, copper is one of the few metals capable of reducing CO_2_ to C_2+_ products due to its optimal binding energy to CO [127, 128, 129, 130]. However, the limited concentration of CO_2_ in the electrolytic bicarbonate system reduces the presence of CO intermediates on the catalyst surface, thereby significantly restricting the Faradaic efficiency and current density of C_2+_ products [131]. Lee et al. [132] designed an electrode with bimetals and dual ionomer to generate C_2+_ products with high efficiency (Figure 11f). Hydrophobic carbon paper was used to create a hydrophobic environment to promote the transport of intermediate CO and inhibit the HER. As a result, FE_C2+_ of 41.6 ± 0.39% was achieved at a current density of 100 mA cm^−2^, which is currently the highest C_2+_ FE value among the reported electrolysis of bicarbonate based on a BPM system.

## 5 Conclusion and Outlook

Considering that CO_2_ typically exists in dilute streams, integrated capture–conversion technologies have been emerging as highly promising approaches for mitigating carbon emissions due to their simplified procedures with economic benefits. The types, mechanisms, merits, and issues of cutting‐edge technologies in this emerging field are particularly focused on by comprehensive summaries and discussions. Coupling the CO_2_ capture and conversion systems in series is a straightforward and simple route, which provides various possibilities since different capture systems (e.g., DAC or CO_2_ capture from marine and flue gas) can be in principle cascaded with any conversion system. However, this type of integration still needs to release CO_2_ from the capture agent, which suffers from high consumption of energy and capture fluids, thus limiting its large‐scale application. As a comparison, the integrated method of directly electrolyzing CO_2_ capture solution is beneficial for saving energy and costs, of which the performance is, however, largely compromised due to the limited CO_2_ available near the active sites. This limitation significantly reduces the current densities and FE due to the promotion of competing HER. In addition, the low‐concentration CO_2_ also impedes the conversion of CO_2_ to C_2+_ products due to insufficient C‐C coupling. Besides, most systems for direct bicarbonate electrolysis still utilize the BPM to provide protons, which definitely increases the ohmic losses and costs of the electrolyzer as well as the possibility of HER occurrence. Besides, the utilization of bifunctional electrocatalysts capable of enriching and converting local CO_2_ can potentially be another effective strategy, which however still suffers from inadequate CO_2_ supply.

To address the common issue of low‐concentration CO_2_ at the electrode/electrolyte interface, reasonable regulation of the local microenvironment can be an effective strategy. For example, the introduction of alkali metal cations (e.g., K^+^, Rb^+^, Cs^+^) can adjust the configuration of the electrochemical double layer, possibly reducing the distance between CO_2_ and the catalyst layer and also inhibiting HER. In addition, the optimal design of GDEs could also increase the local CO_2_ concentration on the catalyst surface. In general, the hydrophobic properties of typical GDEs will limit the diffusion of aqueous capture liquid and thus decayed conversion performance. Therefore, optimizing the GDE structure is expected to enhance the contact between the capture liquid and catalysts, thus overcoming the mass transfer limitation during the electrochemical reduction process. For example, the design of a self‐supporting porous electrode could enhance the diffusion of trapping solutions and meanwhile promote the release of CO_2_ due to the high flow rates. Besides, the design of highly efficient catalysts, which possess excellent ability to enrich and reduce CO_2_, is still one of the attractive research directions in the future. Particularly, the catalysts capable of achieving efficient CO_2_‐to‐C_2+_ conversion are still long‐awaited.

In summary, the integrated capture and electrochemical conversion of low‐concentration CO_2_ from the dilute streams is emerging as a promising yet challenging approach toward efficient carbon utilization in the future. The achievement of integrated CO_2_ capture and conversion technology for industrial applications also necessitates interdisciplinary cooperation, policy support, and technological innovation.

## Conflicts of Interest

The authors declare no conflicts of interest.
